# The Synaptic Gene Study: Design and Methodology to Identify Neurocognitive Markers in Phelan-McDermid Syndrome and *NRXN1* Deletions

**DOI:** 10.3389/fnins.2022.806990

**Published:** 2022-02-18

**Authors:** Jennifer Cooke, Ciara J. Molloy, Antonia San José Cáceres, Thomas Dinneen, Thomas Bourgeron, Declan Murphy, Louise Gallagher, Eva Loth

**Affiliations:** ^1^Forensic and Neurodevelopmental Sciences, Institute of Psychiatry, Psychology & Neuroscience, King’s College London, London, United Kingdom; ^2^Department of Psychiatry, School of Medicine, Trinity College Dublin, Dublin, Ireland; ^3^Fundación para la Investigación Biomédica del Hospital General Universitario Gregorio Marañón, Madrid, Spain; ^4^Biomedical Research Networking Center for Mental Health Network (CIBERSAM), Madrid, Spain; ^5^Department of Neuroscience, Institut Pasteur, Paris, France

**Keywords:** synaptopathies, autism, rare genetic variants, biomarker, Phelan-McDermid syndrome, *NRXN1* deletion

## Abstract

Synaptic gene conditions, i.e., “synaptopathies,” involve disruption to genes expressed at the synapse and account for between 0.5 and 2% of autism cases. They provide a unique entry point to understanding the molecular and biological mechanisms underpinning autism-related phenotypes. Phelan-McDermid Syndrome (PMS, also known as 22q13 deletion syndrome) and *NRXN1* deletions (NRXN1ds) are two synaptopathies associated with autism and related neurodevelopmental disorders (NDDs). PMS often incorporates disruption to the *SHANK3* gene, implicated in excitatory postsynaptic scaffolding, whereas the *NRXN1* gene encodes neurexin-1, a presynaptic cell adhesion protein; both are implicated in *trans*-synaptic signaling in the brain. Around 70% of individuals with PMS and 43–70% of those with NRXN1ds receive a diagnosis of autism, suggesting that alterations in synaptic development may play a crucial role in explaining the aetiology of autism. However, a substantial amount of heterogeneity exists between conditions. Most individuals with PMS have moderate to profound intellectual disability (ID), while those with NRXN1ds have no ID to severe ID. Speech abnormalities are common to both, although appear more severe in PMS. Very little is currently known about the neurocognitive underpinnings of phenotypic presentations in PMS and NRXN1ds. The Synaptic Gene (SynaG) study adopts a gene-first approach and comprehensively assesses these two syndromic forms of autism. The study compliments preclinical efforts within AIMS-2-TRIALS focused on *SHANK3* and *NRXN1*. The aims of the study are to (1) establish the frequency of autism diagnosis and features in individuals with PMS and NRXN1ds, (2) to compare the clinical profile of PMS, NRXN1ds, and individuals with ‘idiopathic’ autism (iASD), (3) to identify mechanistic biomarkers that may account for autistic features and/or heterogeneity in clinical profiles, and (4) investigate the impact of second or multiple genetic hits on heterogeneity in clinical profiles. In the current paper we describe our methodology for phenotyping the sample and our planned comparisons, with information on the necessary adaptations made during the global COVID-19 pandemic. We also describe the demographics of the data collected thus far, including 25 PMS, 36 NRXN1ds, 33 iASD, and 52 NTD participants, and present an interim analysis of autistic features and adaptive functioning.

## Introduction

Autism Spectrum Disorder (ASD)^1^ is characterized by difficulties in communication and social interaction, as well as the presence of restricted and repetitive behaviors and sensory anomalies (American Psychiatric Association [APA], 2013). Currently, around 1 in 54 children are identified as autistic (Maenner et al., 2020), yet still little is known about the links between genomics and clinical outcomes. A significant amount of clinical heterogeneity is observed in autism, with around 70% of people also experiencing other conditions, such as ADHD, anxiety, or epilepsy (Simonoff et al., 2008; Hofvander et al., 2009; Ung et al., 2013; Besag, 2018; Boxhoorn et al., 2018; Lukmanji et al., 2019; Hossain et al., 2020). Despite significant individual variability, autism is a highly heritable condition with strong evidence for the contribution of genetic factors including both inherited and rare *de novo* mutations (Sebat et al., 2007; Buxbaum, 2009; Sanders et al., 2015; Sandin et al., 2017; Bai et al., 2019; Yoon et al., 2021). Although many genes are implicated, these are known to converge on key biological processes, particularly synaptic development and plasticity (Voineagu et al., 2011; De Rubeis et al., 2014; Leblond et al., 2021). Thus, genetic profiles may go some way to explaining the clinical heterogeneity observed in autism and provide key insights into phenotypes linked with specific aetiologies.

Synaptic gene conditions, i.e., “synaptopathies,” involve disruption to genes expressed at the synapse. As some synaptopathies have high penetrance for autism, they provide a unique entry point to better understand the molecular and biological mechanisms underpinning autism-related phenotypes. Such insights are needed to identify new therapeutic targets. They have added to the interest in ‘gene-first’ approaches, which focus on stratifying based on genetic diagnosis, as they open the possibility that new mechanistic treatments, identified in rare synaptopathies, may be applicable for wider sub-groups of individuals with so-called ‘idiopathic’ autism (i.e., without any known genetic rare variants). Hence, the present study was designed to compare two synaptic gene conditions with high penetrance for autism; Phelan-McDermid Syndrome (PMS, also known as 22q13 deletion syndrome, typically including haploinsufficiency of the *SHANK3* gene) and *NRXN1* deletions (NRXN1ds). It is estimated that around 70% of individuals with PMS and 43–70% of individuals with NRXN1ds receive a diagnosis of autism (Soorya et al., 2013; Castronovo et al., 2020). However, despite the reported prevalence of autism within PMS and NRXN1ds, not all individuals receive a diagnosis. In fact, mounting evidence suggests that there may be greater clinical heterogeneity within syndromic or ‘monogenic’ conditions than previously thought (Oberman et al., 2015). For example, both conditions also frequently involve other neurodevelopmental or psychiatric symptoms, including ADHD, psychosis, schizophrenia, bipolar disorder and others (Guilmatre et al., 2014; Kirov et al., 2014; Kolevzon et al., 2019; Cosemans et al., 2020; Vogels et al., 2021). Whilst co-occurring intellectual disability (ID) is common in both synaptopathies, it may vary in severity. Individuals with PMS typically present with moderate to profound ID and delayed or absent speech (Phelan and McDermid, 2012; Soorya et al., 2013; Kolevzon et al., 2014; Oberman et al., 2015). Meanwhile, those with NRXN1ds can present with a more variable cognitive abilities ranging from none to severe ID (Al Shehhi et al., 2019; Cosemans et al., 2020). Heterogeneity in the wider clinical phenotypes of these two conditions may in part be explained by underlying genetic heterogeneity, such that in NRXN1ds, clinical outcome is thought to be attributed to deletion location and possibly size within the *NRXN1* gene (Schaaf et al., 2012; Castronovo et al., 2020; Cosemans et al., 2020). This heterogeneity even within particular monogenic synaptopathies raises both the challenge and opportunity to trace mechanisms by which atypical synaptic transmission, at different developmental levels, brain cells and circuits, may lead to convergent or divergent biological, cognitive and clinical features.

Both *NRXN1* and *SHANK3* genes have an important role in the development of synaptic efficiency, including the maintenance of excitatory and inhibitory *trans*-synaptic signaling. *NRXN1* encodes a presynaptic cell adhesion protein that is implicated in *trans*-synaptic signaling at both excitatory and inhibitory (i.e., glutamatergic and GABA-ergic) synapses within the brain (Reissner et al., 2008, 2013; Anderson et al., 2015; Tong et al., 2017). Meanwhile, *SHANK3* encodes a glutamatergic postsynaptic scaffolding protein that affects the morphology of dendritic spines and synaptic transmission (Gouder et al., 2019; Huang et al., 2019). Initiatives such as the Synaptopathies Consortium, led by Dr. Deepak Srivastava at King’s College London, investigate synaptic biology with a specific focus on glutamatergic synapses, and have shown that stem-cells derived from autistic individuals with *SHANK3* mutations can be linked to early neuronal morphogenesis, suggesting that synaptic development plays a vital role in the pathogenesis of autism (Kathuria et al., 2018). Synaptopathies are thought to affect a fundamental property of brain function, namely the coordinated excitatory and inhibitory activity both at the level of individual neurons (by maintaining appropriate ratios of excitatory vs. inhibitory synaptic inputs), and at the level of global firing patterns within or across networks (Gao and Penzes, 2015). An “imbalance” in excitatory and inhibitory (E/I) transmission has emerged as an influential, albeit somewhat unspecified, theory of a putative pathophysiological mechanism underpinning phenotypic features of autism, such as sensory hypersensitivity, and related neurodevelopmental/neuropsychiatric disorders. At present, animal models exploring *NRXN1* and *SHANK3* expression support E/I imbalance as an index for autistic neural processing (Etherton et al., 2009; Lee et al., 2015, 2017; Sudhof, 2017). E/I imbalance is also suggested from iPSC lines derived from autistic individuals with NRXN1ds, such that derived neurons show upregulated voltage-gated calcium channels and increased Calcium activity (Avazzadeh et al., 2019), as well as increased neuronal excitability (Avazzadeh et al., 2021). This altered neuronal excitability observed in electrophysiological studies brings us closer to identifying neurocognitive markers linked to autistic phenotypes.

It is difficult to directly measure E/I at any level in the living human brain. Although there are now several ligands available to measure glutamate or GABA receptor densities using Positron Emission Tomography (Finnema et al., 2015), the technique is invasive and unsuited for children and/or vulnerable populations. Magnetic Resonance Spectroscopy (MRS) measures, for example glutamate or GABA concentrations in particular brain regions, with the caveat that so-called voxels can span an entire brain structure, thus providing very coarse resolution. Recently, several markers derived from electroencephalography (EEG) have been suggested as ‘proxy’ measures of E/I balance with the potential advantage that EEG is a widely available, relatively inexpensive methodology. Such putative proxy measures include oscillations in the high-frequency gamma and beta ranges, which are thought to reflect, respectively, synchronized fluctuations of the membrane potential of excitatory and inhibitory neurons and involvement of inhibitory interneurons, gated by GABA(A). In addition, neural dynamics, such as the 1/f power law function has been proposed as an index of global network function, while kappa computed dynamically throughout EEG recordings may index critical state change in the brain phases between asynchrony and synchrony (Shew et al., 2009).

Large scale multi-modal natural history studies of rare genetic syndromes are still limited. Although, in the United States, the US Developmental Synaptopathies Consortium^2^, compares people with PMS, Tuberous Sclerosis Complex (TSC1/2), and PTEN Hamartoma Tumor Syndrome.

The Synaptic Gene Study (SynaG) is the first European study that uses a multi-modal approach to address four specific objectives associated with explaining heterogeneity in autism: (1) investigating the frequency of autistic and other neurodevelopmental/neuropsychiatric features within clinically ascertained synaptic gene conditions (PMS and NRXN1ds), (2) determining the extent of shared versus unique autistic features between synaptopathies and those with idiopathic autism, (3) identifying neurocognitive stratification biomarkers, such as E/I imbalance, linked to genotype, and (4) examining the impact of deletion size and additional common or rare genetic factors, such as common genetic burden or additional rare copy number variants (CNV) or single nucleotide variants (SNV), on heterogeneity in the clinical profile of synaptopathies.

SynaG was originally set up as part of EU-AIMS (Loth et al., 2014) and was designed to comprehensively phenotype synaptopathies linked to autism, with a specific focus on PMS. This natural history study aimed to complement pre-clinical work streams using *SHANK3* and *NRNX1* animal models, and human cellular assays focused on synaptopathies that were conducted under the auspices of EU-AIMS to understand the pathophysiological mechanisms underpinning autism-related phenotypes and features (Etherton et al., 2009; Kathuria et al., 2018; Avazzadeh et al., 2021). From this, potential targets for treatment may be highlighted, leading to more personalized approaches to specific neurobiological mechanisms involved in autism.

It is important to clarify at this point that SynaG promotes representation within research and does not seek to pathologise the lived experience of those with synaptopathies or reduce the value of individual variability within autism. The emphasis on biomarker discovery within SynaG serves only to promote treatment choices for those who feel such an approach may improve a person’s quality of life. It is in no way to imply that drug treatment in autism is viewed by authors as required or necessary.

**FIGURE 1:**
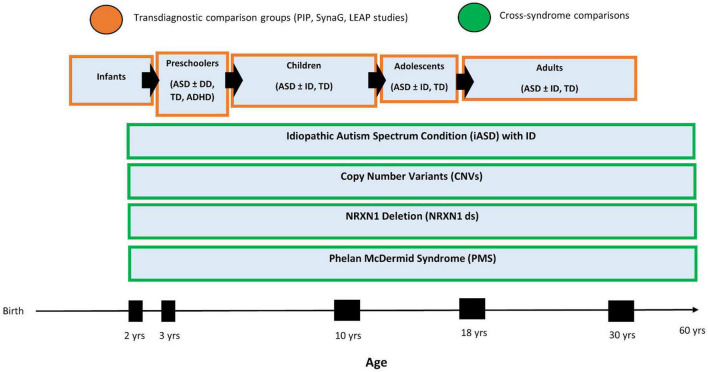
SynaG Study lifespan design and planned comparison groups. The PIP sample includes *n* = 500 participants, the LEAP sample includes *n* = 700, and the SynaG study will include a final sample of *n* = 230 participants.

The NRXN1ds research began as a separate study with a different protocol and was added to SynaG more recently as part of AIMS-2-TRIALS^3^. The study protocols have been aligned across two research sites to allow for comparisons across cohorts within SynaG (see Supplementary Table 1 for SynaG Protocol). Historically, the two studies included each separate comparison groups of, respectively, mental and chronological age-matched participants with typical development. However, SynaG is now embedded within the clinical research portfolio of AIMS-2-TRIALS longitudinal, multidisciplinary research studies. This includes a longitudinal infant sibling studies of 300 babies at high familial likelihood of developing autism or ADHD by virtue of having a first degree family member with either condition, the Preschool Brain Imaging and Behavior Study (PIP), which follows 500 children with autism, ADHD, ID, epilepsy from the age of 3–6 years, and the Longitudinal European Autism Project (LEAP), which follows 730 individuals with autism, mild ID or typical development aged 6–30 years across three time points over 8 years (Loth et al., 2017). Aligning the SynaG protocol with PIP and LEAP, depending on the participant’s age or ability level, allows us to make use of a large number of comparison participants with typical development, ID or ASD. In particular, it enables us to adopt a “normative modeling” approach across experimental measures (i.e., cognition, eye-tracking, EEG, and MRI indices) which is akin to normative growth charts frequently used by pediatricians to assess a child’s height or weight (Marquand et al., 2019). This approach first involves deriving “standardized” scores of different age groups in terms of say, regional cortical thickness, and then ascertains how far scores of a particular individual (with PMS and NRXN1ds) differ from expectations based on the participant’s age, mental age, sex and/or other variables (see Figure 1). This approach is well-suited to make predictions about an *individual* (as opposed to group-level inferences), which is critical for the clinical use of biomarkers.

In addition, some aspects of the SynaG protocol are also aligned with the Developmental Synaptopathies Consortiums natural history study of PMS protocol (led by Alexander Kolevzon at Icahn School of Medicine at Mount Sinai^4^) for data pooling and replication, which is critical when working with rare disorders.

The current paper sets out the design and methodology for the SynaG study. Given the prevalence of mild to profound ID within our genetic populations of interest, we also include strategies used to facilitate engagement, in addition to the necessary adaptations made during the global COVID-19 pandemic. As data acquisition is ongoing, we present sample demographics and task completion numbers for each modality at the time of writing (August 2021).

## Materials and Methods

### Design

The study adopts a cross-syndrome design including two genetically defined cohorts: PMS, NRXN1ds, and two comparison groups of idiopathic autism (iASD) (with ID) and neurotypical development (NTD). The individual and both biological parents are invited to take part in the study so that the heritability of certain genetic features and clinical characteristics associated with autism can be assessed.

### Sample

The objective is to assess 50 PMS, 50 NRXN1ds, 30 iASD with ID, 100 typically developing (NTD) matched controls (*N* = 230). Sample sizes for each participant group were determined with consideration of data collection from the existing EU-AIMS LEAP study and with the feasibility of data collection in mind, given that both PMS and NRXN1ds are rare conditions. Participants are recruited and tested at two different sites, King’s College London (KCL) and Trinity College Dublin (TCD). Written consent or assent is obtained from participants, and from a parent/legal guardian where the participant is below the age of 16 (United Kingdom) or 18 years (Ireland) and where participants lack mental capacity to consent for themselves, for example in cases of profound ID. Where necessary, mental capacity is assessed by trained members of the research team in conjunction with parent/legal guardian appraisal. Ethical approval was obtained from St. James’ Hospital/The Adelaide and Meath National Children’s Hospital (REC ref: 2015/03/01) and the St. James’ Hospital/Tallaght University Hospital Joint Research Ethics Committee [REC ref: 2019-09 List 35 (10)] at TCD, and the UK Health Research Authority via the Queens Square Research Ethics Committee (REC ref: 15/LO/0305) and the South London and Maudsley NHS Foundation Trust (SLaM ref: CSA/17/001) at KCL.

Mental age-matched NTD and iASD comparison groups have been recruited to account for the impact of severe to profound ID on phenotypic presentation in PMS. As severe ID is less prevalent within NRXN1ds, chronological age and gender-matched NTD participants have also been recruited to serve as an adequate comparison of age and ability level for this group. As a result, the current NTD group includes a wide range of ages (18 months – 54 years) and developmental levels (i.e., toddlerhood – adulthood). To ensure a fair comparison of ability level between our affected groups (PMS, NRXN1ds, and iASD) and typical development we have categorized our current NTD group as follows for this paper: NTD-PMS, referring to those younger NTD participants more closely representing the ability level of PMS and iASD participants with severe to profound ID, and NTD-NRXN, who are age and gender-matched to NRXN1ds participants and more closely represent the ability level of this genetic group. Current sample demographics, including chronological age (CA) and non-verbal mental age (NVMA) are presented in Table 1.

**TABLE 1:** Current sample demographics including both KCL and TCD sites.

		PMS	NRXN1ds	iASD	NTD-PMS	NTD-NRXN
Total *N*		25	36	33	30	22
Gender (f:m)		12:13	17:19	4:29	13:17	9:13
Autism dx (%)		32	33	100	0	0

Non-verbal mental age (NVMA) in years*	***n***	**21**	**20**	**28**	**28**	**21**

	Mdn	1.54	32.51	3.33	4.59	35.23
	IQR	10.02	27.80	2.31	2.38	25.05
	Min–max	0.50–47.32	7.75–51.68	0.75*–10.44	1.46–7.19	9.49–55.70

**Chronological age (CA) in years**	***n***	**25**	**36**	**33**	**30**	**22**

	Mdn	6.56	20.56	6.80	4.24	33.70
	IQR	5.66	25.49	6.04	2.84	26.80
	Min–max	2.00–47.32	2.25–52.20	2.86–19.42	1.57–6.92	10.20–53.62

*NVMA was estimated as the average age equivalent for the visual reception and fine motor domains for the MSEL, and as (chronological age x perceptual reasoning index)/100 for the WASI-II. The accuracy of these estimates may vary in cases of severe to profound ID where the precision of behavioral assessment decreases.

Crucially, our current iASD comparison group includes only individuals with co-occurring ID to control for severe to profound ID in our PMS group. Although, as outlined previously, greatly enriched comparison groups will ultimately be generated by combining samples from several studies within the AIMS-2-TRIALS research portfolio.

### Inclusion and Exclusion Criteria

Participants with PMS or NRXN1ds are included if they have clinically confirmed CNVs via existing clinical genetics reports. For PMS, we allow both individuals with and without a *SHANK3* deletion. CNVs are confirmed using aCGH or SNP array from accredited clinical genetics services in the United Kingdom or Ireland, or through SNP array by our AIMS-2-TRIALS consortium partners at Institut Pasteur in Paris, France. iASD is defined in our study as first diagnosis autism in the absence of any known genetic syndrome, at the time of study enrollment, which is then confirmed by consortium partners at Institut Pasteur. iASD participants are in receipt of a formal diagnosis of Autism Spectrum Disorder (ASD) according to either the Diagnostic and Statistical Manual for Mental Disorders (DSM) or the International Classifications of Diseases (ICD) criteria (American Psychiatric Association [APA], 2000, 2013; World Health Organization [WHO], 2013).

For PMS, NRXN1ds, and iASD cohorts, additional co-occurring neurodevelopmental or psychiatric conditions are allowed (except for psychosis in the iASD group), given the prevalence of comorbidity and because ascertaining neurodevelopmental/neuropsychiatric features is a key objective of the study. Participants on stable medication (minimum of 8 weeks at entrance point) are also eligible for the study (in all groups, including NTD). Exclusion criteria for the NTD group is a formal diagnosis of any neurodevelopmental or neuropsychiatric condition and/or the presence of any professional concerns or investigations around development, i.e., from teachers or health professionals.

Noteworthy, as NRXN1ds can be inherited, any biological parent subsequently identified as a carrier of a NRXN1ds following their child’s genetic diagnosis are also included in the study as participants, but often have no known diagnosis of a neurodevelopmental condition.

### Study Visit Structure

Participating families are invited to attend 2-day visits at the research institute. Day 1 consists of clinical and cognitive phenotyping assessments, parent interviews, eye-tracking tasks, EEG recording, and biosampling. Day 2 typically involves the MRI scanning protocol and the completion of outstanding tasks. The visit structure broadly follows that of the EU-AIMS LEAP and is described in more depth in a previous publication (Loth et al., 2017).

Participants are allocated to testing schedules based on their estimated NVMA as ascertained during screening, and therefore complete measures suitable to their level of non-verbal ability. Consequently, certain tasks/measures are not completed in all groups. All tasks in the study protocol are considered optional, thus, families can consult with the research team and choose to opt-out of certain tasks, depending on a participant’s individual needs and ability level. Support to engage with all aspects of the study protocol is provided by the research team and tailored to each family’s individual needs (more information is provided in Supplementary Box 1). For a full list of SynaG testing schedules and measures described in the methods section please see Supplementary Table 1.

### Frequency of Autism and Neurodevelopmental/Neuropsychiatric Features

To address SynaG study objectives 1 and 2, a range of clinical and cognitive assessments, and self or parent-report questionnaires are used to comprehensively phenotype our clinically ascertained synaptopathies and comparison groups.

#### Characterization of Autism

Autistic features are assessed in our genetic cohorts and confirmed for our iASD group using the Autism Diagnostic Observation Schedule 2 (ADOS-2) and the Autism Diagnostic Interview-Revised (ADI-R) (Lord et al., 2012; Rutter et al., 2012). Autism diagnostic assessments are not completed with NTD participants. However, parents of all children are asked to complete the Social Communication Questionnaire - Lifetime (SCQ-Lifetime) and the Children’s Routines Inventory - Revised (CRI-R) to dimensionally assess level of social communication symptoms and restricted and repetitive behaviors, across all groups, including NTD. Autism diagnostic assessments are also not collected in parent NRXN1ds cases; instead, the Autism Quotient, Social Responsiveness Scale, Adulthood Routines Inventory- Revised (ARI-R) and SCQ-L are collected to measure autism features.

#### Further Neurodevelopmental and Neuropsychiatric Characterization

Temperament, behavioral disturbances, and motor coordination are assessed using a range of validated parent or self-report questionnaires depending on the age and developmental level of the individual. Attention Deficit Hyperactivity Disorder (ADHD) and sensory dysregulation are also assessed using standardized parent or self-report measures (McIntosh et al., 1999). For a full list of named questionnaires see Supplementary Table 1.

#### Cognitive and Adaptive Functioning

Both verbal and non-verbal mental ages are established for all participants using an assessment suitable to the individual’s ability and level of language, which is estimated by researchers through parent-report and initial observations of the child at the visit. Assessment measures include the Mullens Scales of Early Learning (MSEL), the British Picture Vocabulary Scale: Third Edition (BPVS) and Raven’s Colored Progressive Matrices (CPM), the British Ability Scales (BAS), and the Wechsler Abbreviated Scale of Intelligence - Second Edition (WASI–II) (see Supplementary Figure 1 for how assessments are assigned in each schedule). Daily adaptive functioning, including social adaptive functioning, is assessed for all participants using the Vineland Adaptive Behavior Scales second edition (Vineland-II) parent interview format (Sparrow et al., 2005). Global functioning and coordination disorders are additionally screened.

#### Medical and Family Background

In order to establish a comprehensive phenotypic profile for each participant, a full medical and psychiatric history is recorded. Early environmental factors, such as maternal depression and drug, alcohol or medication use during pregnancy, are also recorded as they have been linked to neurodevelopmental outcomes in previous work (Kim et al., 2019). Sleep disorders are commonly reported in neurodevelopmental conditions (Devnani and Hegde, 2015); therefore, we additionally measure sleep habits for all participants.

### Parent Phenotyping

Parents are screened for autistic features, ADHD, anxiety, and depression to better understand the potential role of family background on clinical heterogeneity. These measures are collected in both biological parents where possible and biological samples (bloods or saliva, and hair) are also collected to further assess genetic heritability in our populations of interest.

### Social Cognition

To assess social cognition in younger and non-verbal participants, two play-based tasks probe for social attention and Theory of Mind (ToM), namely the Social Orienting task and the Penny Hiding Game (Dawson et al., 2004; San Jose Caceres et al., 2014). Additionally, an unstructured observation of parent-child play is recorded to capture a naturalistic interaction in the absence of any task demands. Previous research has linked reduced social attention and limited spontaneous initiation of social interaction with a caregiver to early development to autism (Devnani and Hegde, 2015).

### Identification of Neurocognitive Stratification Biomarkers

To address objective 3 of the SynaG study we leverage eye-tracking, EEG and MRI metrics to uncover neurocognitive markers of autism linked to specific genotypes. Of particular interest are proxy markers of synaptic transmission, such as E/I imbalance, which may represent an aetiological marker for autism in these groups.

#### Eye-Tracking

Eye-tracking is used as an objective measure of visual attention, including social attention, through assessment of eye-movements and gaze patterns. Reduced motivation for social engagement and differences in attention to social content has been associated with autism (Chevallier et al., 2012). We will assess if eye-tracking measures of visual attention can be leveraged as a potential neurocognitive marker of autism associated synaptopathies. A battery of tasks were chosen based on their suitability across a range of ages and ability levels, including young children and those with ID, which only require a participant to look at and explore stimuli displayed on the screen. The battery includes a set of static and dynamic social stimuli, which are used to measure spontaneous social attention. A biological motion task using point light display stimuli to measure preference for biological motion or non-biological (scrambled) motion (Annaz et al., 2012). A third task, the implicit false belief task, is included as a measure theory of mind, which is the ability of a person to attribute mental states to oneself and another person. In this task we assess gaze patterns during a short video to determine if participants can represent the person in the video’s false belief. Finally, the gap overlap task (Landry and Bryson, 2004; Elsabbagh et al., 2013) is a gaze contingent paradigm that measures visual attention disengagement from a central to a peripheral stimulus, which is thought to be related to top-down attentional control. Attentional disengagement has been shown to be a good measure of the development and maturation of visual attention. Differences in the time taken to disengage and shift attention to another stimulus has also been suggested to reflect differences in processing of local and global information, often reported as a feature of early visual attentional processing in autism.

#### Electroencephalography

Electroencephalography provides an index of neural responsiveness and synchronization that reflects neurochemical changes at the level of the synapse. Altered synaptic development and functionality are pre-existing features of the synaptopathies within SynaG. Atypical attentional processing and variation in network level neural firing patterns, such as E/I imbalance, can be tracked using EEG and have been linked to autistic brain processing in the literature as previously described (Dickinson et al., 2016; Bruining et al., 2020). Therefore, we utilize EEG methodology for biomarker detection in SynaG as a way to identify neural signatures that can be linked to the genotype of our synaptopathies of interest (PMS and NRXNds).

Two EEG metrics, namely event-related potentials (ERPs) and event-related oscillations (EROs) are implemented to stratify groups. To assess group and individual differences we firstly explore pre-attentive ERP responses associated with change detection (mismatch negativity, MMN) and novelty detection (P3a), thought to reflect the organization of attention-related processes within the brain (Kujala et al., 2013; Fitzgerald and Todd, 2020). Secondly, we use frequency-based analyses of beta, theta and alpha bands to explore neural synchronization during the passive viewing of social and non-social videos. Functional connectivity analysis will also be used to examine short and long-range synchronization (i.e., connectivity) within and between different brain networks. Lastly, we use an auditory gamma task to probe for power in the gamma frequency, phase-locking factor, and power in the stimulation frequency (Rojas and Wilson, 2014). Power within the gamma frequency across all tasks will be examined as a proxy marker for E/I imbalance given the specific relevance to autism and synaptopathies (Rojas and Wilson, 2014; Kolesnik et al., 2019).

#### Magnetic Resonance Imaging

Magnetic resonance imaging is used to characterize brain structure and connectivity across individuals and groups within SynaG. We aim to identify system-level biomarkers relating to genotype that may explain variability observed at the phenotypic-level. High resolution anatomical T1-weighted scans are acquired to investigate structural biomarkers of brain volume, cortical thickness and surface area. Whilst, diffusion tensor imaging (DTI) is used to analyze white matter structural architecture. E/I imbalance is probed using magnetic resonance spectroscopy (MRS), whereby we measure absolute metabolite concentrations, specifically Glutamate and GABA, in the anterior cingulate cortex, concordant with recent findings in autism (Horder et al., 2018; Ajram et al., 2019), and the known genetic basis of our two synaptopathies of interest (PMS and NRXNds).

Several procedures have been put in place to facilitate MRI scanning at KCL, particularly with young children and individuals with severe or profound ID. At KCL, a space narrative has been introduced, which includes a space-themed tent that can be erected to partially cover the scanner, and ‘space friends,’ which are MRI-safe soft characters that can be attached to the space tent using Velcro (see Supplementary Box 1 and Supplementary Figure 2). KCL have also recently introduced a sleep scanning protocol whereby participants can attend the scan session in the evening for an unlimited time with their parents or carers. Furthermore, participants may also receive melatonin as an aid to sleep prior to entering the scanner, where all relevant medical checks have been made. The procedure was introduced after consultation with a number of participating families who felt their children would not be able to remain still in the MRI scanner whilst awake.

### Biological Samples

To address objective 4, blood and saliva samples are collected to examine how genetic variation may relate to the heterogeneity in clinical phenotype, and how this may potentially relate to altered underlying function at the cellular level. Blood, saliva, hair and skin biopsy samples are collected from study participants, their parents, and where appropriate, a sibling.

#### Genomics

DNA is extracted from blood and saliva samples. Saliva samples are used for a SNP array using Illumina NovaSeq 6000 platform. Blood samples are used for whole-genome sequencing which allows for CNV breakpoint mapping and also the identification of common and rare variation elsewhere in the genome. Following sequencing, quality control and variant calling/validation will be performed following methods outlined by AIMS-2-TRIALS LEAP WGS. We also confirm *NRXN1* and *SHANK3* deletion size and the CNV’s breakpoints to better understand their influence on severity of clinical characteristics, as well as behavior and cognition. Next, we test whether a participant carries one or more additional CNVs associated with neurodevelopmental conditions at the same locus (a double hit), or at another locus as defined by Kendall et al. (2019). Finally, we examine the burden of other hits, i.e., additional, putatively loss-of-function, deleterious genetic variants, or primary hits, i.e., clinically validated genetic variants associated with neurodevelopmental disorders. It is hoped that understanding the contribution of this other hit burden to the observed clinical, behavioral, neurological and physical phenotypes may improve our understanding of the heterogeneity in clinical and cognitive outcomes in NRXN1ds and PMS.

#### Development of Induced Pluripotent Stem Cells

Hair samples (keratinocytes) are used to generate induced pluripotent stem cells (iPSCs) of selected volunteers with a particular phenotypic (e.g., ASD vs. no-ASD) and/or genomic profile (e.g., *SHANK3* plus double-hit, or a particular genetic background) following a protocol developed by Professor Jack Price and colleagues at KCL (Warre-Cornish et al., 2020). Skin biopsy samples collected from autistic NRXN1ds participants have previously been used to derive iPSC lines to examine neuronal function (Avazzadeh et al., 2019, 2021). Human iPSC studies derived from individuals with *SHANK3* and *NRXN1* deletions may reveal important information about differences in neuronal activity and function, which may benefit our understanding of clinical phenotype, as well as the development and testing of targeted treatments.

### Planned Analyses

To address aim 1 of the SynaG study, we will conduct traditional mean-group comparisons to establish whether our synaptopathies differ on average from each other with respect to the frequency of autistic and associated neurodevelopmental/neuropsychiatric features.

To address aim 2, we will assess whether mean-group differences exist between our synaptopathies and an idiopathic autistic group (i.e., autism without a known genetic association) and also a typically developing group (i.e., those without diagnosis or suspicions of neurodevelopmental conditions).

However, mean group differences are not well suited to understand individual variability within group (Dumas et al., 2021). Therefore, broadly following the approach used in standardized assessment tools, we aim to standardize scores on each experimental measure by creating ‘norms’ based on the larger data sets available via the AIMS-2-TRIALS PIP and LEAP cohorts. The approach enables us to derive ‘scaled’ scores relative to each person’s chronological age and/or mental age (developmental level). This is a particularly poignant approach for research into rare genetic conditions because it directly addresses the common issue of small sample bias in group-level comparisons and enables us to make predictions about individuals. That is, given the inherent rarity of such genetic conditions it can be difficult to generalize group level findings to an entire affected population. With a normative modeling approach (also outlined previously), we will be able to compare individual performance of those with synaptopathies against an extensive comparison population that spans several stages of human development from toddlerhood to adulthood, including those with neurodevelopmental conditions.

Assessing individual performance in this way affords us the opportunity to identify variations in the developmental trajectories of those with synaptopathies and make predictions about possible future outcomes, which is key to improving our understanding of the natural history of neurodevelopmental conditions, but also important for managing ongoing support or treatments for these individuals. Additionally, the introduction of such an extensive comparison group will also be used to enrich our between-group comparisons (particularly where our current groups may not be matched on sex, age or developmental level).

Aim 3 will be addressed using the data modeling and comparison approach previously outlined, so that we may identify neurocognitive, functional, and/or structural biomarkers specific to our synaptopathies of interest.

Aim 4 will involve observing genotype-phenotype correlations to investigate in what ways phenotypic presentation may be related to genetic profile in our synaptopathies of interest and idiopathic autistic groups. Genetic profiling in SynaG will include location and size of deletion, and examination of common genetic burden or additional rare CNVs or SNVs and their impact on clinical phenotype.

### Facilitating Study Engagement

Several strategies are implemented to increase chances that participants with PMS and NRXN1ds (i.e., mild to profound ID, and complex needs) are able to complete the various experimental measures (see Supplementary Box 1 for description of strategies). Researchers work closely with parents to understand the specific needs of each child, which may include limited attention span, difficulties sitting still for long periods of time, or sensory difficulties that could impact toleration of the EEG cap or the noise of the MRI scans. The approaches used in SynaG are a combination of expertise within the research team, consultation with participating families, and ongoing discussions and ideas drawn from research groups and publications in the area (Webb et al., 2015; DiStefano et al., 2019). Specific credit should go to the MIND Institute, Sacramento, CA, United States, for their advice in conducting MRI sleep scans (Nordahl et al., 2008, 2016; Amaral et al., 2017).

### COVID-19 Adaptations – Remote Testing Procedures

In March 2020 all face-to-face testing for the study was suspended due to COVID-19 restrictions. In response to these restrictions, and due to uncertainties regarding the resumption of in-person testing, both research groups adapted the study protocol to continue collection of some assessments remotely. Specifically, our remote testing procedure includes scheduling ADI-R, Vineland-II and family history interviews as online video call interviews with parents instead of during visits to our neurocognition lab. All parents and self-reporting participants are also now sent a link through email and asked to complete questionnaires online at home. Finally, saliva sample kits are sent by post to family homes and samples are collected from participants and their parents. These samples are then returned by post. ADOS-2, ET, EEG, and MRI data collection were all suspended as of March 2020. The research labs are now reopening for face-to-face testing allowing us to continue with our protocol as of September 2021. Dates and timelines for all testing have been recorded to account for any delay between remote and face-to-face assessment.

## Results

### Interim Analyses – Sample Demographics

25 PMS, 36 NRXN1ds (including previous and SynaG studies), 33 iASD, 30 NTD-PMS, and 22 NTD-NRXN participants have been enrolled into the SynaG study so far (*N* = 146) (see Table 1). Median chronological age (CA) was 6.56 years for PMS (2.00–47.32 years), 20.56 years for NRXN1ds (2.25–52.20 years), 6.80 years for iASD (2.86–19.42 years), 4.24 years for NTD-PMS (1.57–6.92 years), and 33.70 years for NTD-NRXN (10.20–53.62 years) (see Table 1). A Kruskal–Wallis test confirmed that CA was different across groups [*H*(146,4) = 70.305, *p* < 0.001]. *Post hoc* (Dunn’s) pairwise comparisons with Bonferroni correction confirmed that our current PMS (*p* < 0.01, *p* < 0.001), iASD (*p*s < 0.001), and NTD-PMS (*p*s < 0.001) groups are younger on average than the NRXN1ds and NTD-NRXN groups respectively, in accordance with the comparison sample matching procedures outlined under Section “Sample.” Furthermore, NTD-PMS participants are younger on average than both PMS and iASD participants (*p*s < 0.05). NRXN1ds and NTD-NRXN groups are currently matched on CA as predicted (*p* = 1). PMS and iASD groups are also currently matched on CA (*p* = 1).

A higher proportion of males to females (29:4) was observed only in the iASD group, consistent with existing literature on autism (see Table 1) (Werling and Geschwind, 2013; Loomes et al., 2017). Prior to entering the study, 32% of PMS and 33% of NRXN1ds were in receipt of a formal diagnosis of autism (see Table 1).

Differences in non-verbal mental age (NVMA) were present across groups as anticipated [*H*(118,4) = 80.446, *p* < 0.001]. *Post hoc* pairwise comparisons with correction revealed that on average, NRXN1ds (mdn = 32.51) and NTD-NRXN (mdn = 35.23) participants had a higher NVMA than PMS (mdn = 1.54, *p*s < 0.001), iASD (mdn = 3.33, *p*s < 0.001), and NTD-PMS participants (mdn = 4.59; *p*s < 0.001) (see Table 1). PMS, iASD, and NTD-PMS groups are currently matched on NVMA (*p*s > 0.1), in accordance with comparison sample matching procedures. NRXN1ds and NTD-NRXN groups are also currently matched on NVMA (*p* = 1).

**FIGURE 2:**
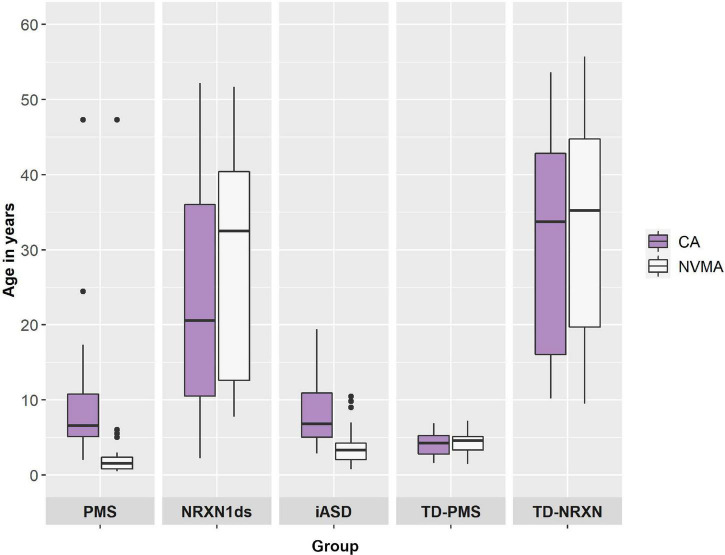
Chronological age (CA) and non-verbal mental age (NVMA) comparisons by diagnostic group.

Discrepancy between CA and NVMA as a general indication of developmental delay was assessed in each group using paired samples Wilcoxon signed rank tests (for contributing group *n*’s please see *n*’s for NVMA in Table 1). On average, developmental delay (i.e., NVMA significantly lower that CA) was present for PMS (*Z* = 3.920, *p* < 0.001), iASD (*Z* = 4.509, *p* < 0.001), and NRXN1ds groups (*Z* = 3.061, *p* < 0.01), with no difference for NTD-PMS (*Z* = −0.775, *p* = 0.439) and NTD-NRXN (*Z* = −1.269, *p* = 0.204) comparison groups (see Figure 2).

### Interim Analyses – Task Completion Numbers

Supplementary Table 2 provides a full breakdown of task completion numbers by group. Autistic features have been assessed in between 84–100% of PMS, 19–28% of NRXN1ds, and 85–91% of iASD participants thus far. Cognitive functioning has so far been assessed in 88% of PMS, 61% of NRXN1ds, 91% of iASD, 100% of NTD-PMS, and 95% of NTD-NRXN participants. Additionally, 58% of NRXN1ds and 95% of NTD-NRXN participants have been further assessed using the CANTAB. Adaptive functioning has been assessed in 96% of PMS, 22% of NRXN1ds, 88% of iASD, and 93% of NTD-PMS participants.

At the KCL site, between 72–80% of PMS, 76–91% of iASD, and 97–100% of NTD-PMS participants have completed behavioral measures of social cognition, specifically the Social Orienting task and Parent-Child Interaction. Only 28% of PMS participants have completed the Penny-Hiding game task as the majority lacked the fine motor skill necessary to complete all aspects of the game for assessment when attempted. At TCD, 69% of NRXN1ds participants have completed interviews/questionnaires probing for comorbidities (i.e., the DAWBA).

Neurocognitive measures such as ET and EEG have thus far been completed by between 72–84% of PMS, 6–8% of NRXN1ds, 48–70% of iASD, 73–100% of NTD-PMS, and 50–100% of NTD-NRXN participants. MRI completion is currently at 16% in PMS, 47% in NRXN1ds, 18% in iASD, 20% in NTD-PMS, and 77% in NTD-NRXN. Biosamples including bloods, saliva, hair, and skin biopsy (only at TCD), have thus far been completed by between 60–84% of PMS, 6–31% of NRXN1ds, 9–79% of iASD, and 3–77% of NTD-PMS participants.

Please be aware that due to COVID-19 restrictions, several NRXN1ds participants and a smaller number of PMS participants, are still awaiting a face-to-face research visit. Therefore, the acquisition of measures such as ET, EEG, MRI scanning, and biosample collection have been severely impacted. Despite this, parent interviews and online questionnaires have been completed remotely wherever possible. All aspects of the SynaG research protocol are now to be resumed (as of September 2021).

Also of note is the optional nature of the SynaG protocol, whereby parents can select for their child to opt-out of aspects of the study protocol they feel will be too challenging for their child. The types of assessment frequently abandoned and why are to be explored within a subsequent publication. As outlined in more detail elsewhere (Supplementary Box 1), the research team work closely with all families to facilitate participation where possible and support the best interests of the child.

## Discussion

The SynaG study adopts a gene-first approach to autism that aims to understand the impact of specific genetic susceptibility factors on neurocognitive and behavioral development. Two specific synaptic gene conditions (“synaptopathies”) with a high penetrance for autism, namely PMS and NRXN1ds, are considered. The study is an important step toward explaining heterogeneity within autism in a biologically meaningful way. To our knowledge, this is the first European study that compares these two specific CNVs on a comprehensive multi-disciplinary protocol.

In order to comprehensively phenotype individuals, a variety of clinical and cognitive measures are collected, ranging from parent questionnaires and behavioral assessments to participant EEG recordings and MRI scans. Study recruitment is ongoing and so the current paper serves to showcase what progress has been made from the inception of the SynaG study in 2014 until the present day, as well as highlight that in spite of many challenges (e.g., individuals with significant intellectual disability, and the current COVID 19 pandemic) it is possible to deeply phenotype rare and complex populations.

### Current Sample

The current study sample, including comparison groups, consists of a broad range of ages and ability levels. Around one third of our PMS and NRXN1ds groups (32 and 33%, respectively) were in receipt of a formal diagnosis of autism spectrum disorder prior to entering the study. Crucially, half of our current PMS cohort are under the age of 6 years, which may explain why we observe a lower number of autism cases than previously reported at this point (Soorya et al., 2013). That is to say, that autistic features may have yet to manifest in a way that warrants further assessment for these individuals. Moreover, it is possible that medical concerns/conditions take priority for assessment and treatment in the early stages of development. As individuals with PMS commonly present with multiple and profound disabilities, autistic features become part of a complex clinical presentation that requires skilled formulation and assessment. There is some evidence to suggest that general developmental delay may inflate the likelihood of autism diagnosis within PMS (Oberman et al., 2015). Furthermore, it has been found that the autistic phenotype may be influenced by the contribution of both deleted and preserved genes within the *SHANK3* region (Oberman et al., 2015). In order to address some of these challenges, Soorya et al. (2018) have published a framework for the assessment of individuals with rare genetic disorders, as a guide for professionals.

For our NRXN1ds group, it should be noted that parent NRXN1ds carriers ascertained thus far do not frequently have pre-existing diagnoses of autism and so may contribute to the current levels of autism we observe in NRXN1ds being on the lower percentage of reported in the literature. Ascertainment and inclusion criteria may contribute to the variability in frequency of reported autism diagnosis across studies.

### Study Progress

Task completion numbers are promising given the extensive nature of the study protocol and the complex needs of our sample. The encouraging level of engagement from families may be due to the carefully designed and accessible protocol, as well as training and experience of the researchers in working with populations with ID, and attention to environmental modifications. The majority of participants are engaging well with behavioral assessments of autism and cognitive functioning. Parents/guardians are equally engaging well with interviews about their child’s early development and adaptive functioning. Acquisition of blood samples has been a particular challenge in the iASD group due to a high prevalence of tactile sensitivity. For our more recently added NRXN1ds cohort, task completion numbers are currently low due to face-to-face assessments being suspended during the COVID-19 pandemic. Furthermore, several PMS and iASD participants are currently awaiting MRI scanning under our melatonin protocol as a result of COVID-19 related suspension. With the resumption of face-to-face research visits as of October 2021, specific focus will be given to improving task completion numbers for MRI and biosampling measures. In order to achieve this, the research team will continue to work closely with families to implement supportive behavioral strategies, as well as offering our sleep scanning MRI protocol with melatonin.

Until now, the SynaG study has cataloged a sample of individuals with synaptic conditions, along with iASD and NTD comparison groups, but the research is ongoing. Data collection in rare genetic syndromes is challenging given the inherent rarity of cases, with the prevalence of co-occurring ID and complex needs also acting as a barrier to accessing extensive research protocols, such as that within SynaG. However, with the upscaling of remote testing during the global COVID-19 pandemic, the introduction of MRI scanning with melatonin, and the continued utilization of supportive behavioral strategies, we are confident that we will reach our proposed recruitment targets.

### Future Study Plans

Much of what is currently known about the links between synaptopathies and autism comes from pre-clinical studies that are far removed from the clinical outcomes experienced by autistic individuals. SynaG sets a precedent for translational neuroscience by combining with the prior knowledge-base of preclinical studies to put individuals with synaptic gene conditions at the heart of the research. What is more, current clinical trials within the wider AIMS-2-TRIALS consortium are focused on targeting GABA receptors to address E/I imbalance with respect to improving social adaptive functioning in autistic children and adolescents. By partnering with initiatives such as the Synaptopathies Consortium and the wider AIMS-2-TRIALS consortium, we hope to uncover neural markers linked to synaptic functioning that may serve as potential treatment targets in future. Biomarker discovery may also inform the effectiveness of non-drug-based interventions that could improve quality of life.

Future plans for the SynaG study involve expansion to a longitudinal design whereby developmental trajectories and outcomes can be mapped in relation to iASD. One substantial benefit of being part of the AIMS-2-TRIALS consortium is the possibility of comparing our rare samples against the largest longitudinal cohort of autistic individuals ever recorded in LEAP (Loth et al., 2017). In this way, we can identify whether individuals with synaptic gene conditions follow similar developmental trajectories to those with iASD, as well as how and where they may deviate. The approach may be extremely informative in relation to explaining heterogeneity as the inherent rarity of these conditions translates to small research samples and an inability to make accurate population level inferences. What is more, the ability to associate unique developmental trajectories with specific genotypes takes us one step further in understanding and predicting clinical outcomes in autism. Collaboration between research teams is crucial for generating large samples in rare disorders that allow for population level inference to be drawn accurately. SynaG will continue to actively collaborate with the Developmental Synaptopathies Consortium and remains open to collaboration with other research teams around the world working in the area of synaptic gene conditions.

### Conclusion

The SynaG study takes a gene-first approach to explaining heterogeneity in autism, focusing on PMS and NRXN1ds. The study compliments existing pre-clinical work looking at the downstream effects of alterations to *SHANK3* and *NRXN1* genes. Biomarker discovery in synaptopathies, such as PMS and NRXN1ds, is a promising area of research for the identification of potential treatment targets and more personalized treatment approaches.

## Data Availability Statement

The original contributions presented in the study are included in the article/Supplementary Material, further inquiries can be directed to the corresponding authors.

## Ethics Statement

The studies involving human participants were reviewed and approved by St. James’ Hospital/The Adelaide and Meath National Children’s Hospital (REC ref: 2015/03/01) and the St. James’ Hospital/Tallaght University Hospital Joint Research Ethics Committee [REC ref: 2019-09 List 35 (10)] at Trinity College Dublin, and the UK Health Research Authority via the Queens Square Research Ethics Committee (REC ref: 15/LO/0305) and the South London and Maudsley NHS Foundation Trust (SLaM ref: CSA/17/001) at King’s College London. Written informed consent to participate in this study was provided by the participants’ legal guardian/next of kin.

## Author Contributions

EL, TB, and DM were involved in the conception of the original study design. EL and ASJC were responsible for initiating the study at the KCL site. LG, EL, CM, and JC were involved in expanding the study at the TCD site. JC and CM compiled and analyzed the data and wrote the article in full. ASJC, TD, EL, and LG contributed to the composition and drafting of the article. JC, ASJC, and CM have made significant contributions to data collection for this study. All authors approved the final version to be published.

## Author Disclaimer

Any views expressed are those of the author(s) and not necessarily those of the funders.

## Conflict of Interest

ASJC has worked as a consultant in several different projects for Servier and Roche. The remaining authors declare that the research was conducted in the absence of any commercial or financial relationships that could be construed as a potential conflict of interest. The reviewer EB declared a past co-authorship with the authors TB and LG to the handling editor.

## Publisher’s Note

All claims expressed in this article are solely those of the authors and do not necessarily represent those of their affiliated organizations, or those of the publisher, the editors and the reviewers. Any product that may be evaluated in this article, or claim that may be made by its manufacturer, is not guaranteed or endorsed by the publisher.

## Footnotes

1 Henceforth referred to as autism in accordance with the express wishes of the autistic community.

2
https://www1.rarediseasesnetwork.org/cms/dsc

3
https://www.aims-2-trials.eu/

4
https://www1.rarediseasesnetwork.org/cms/dsc/Get-Involved/Studies/7902
