# Supplementary material for: The Synaptic Gene Study: Design and Methodology to Identify Neurocognitive Markers in Phelan-McDermid Syndrome and NRXN1 Deletions
Source: Front Neurosci. 2022 Feb 18;16:806990. doi: 10.3389/fnins.2022.806990 (PMC8894872; doi:10.3389/fnins.2022.806990)
Supplement: Supplementary file 1 [file Table_1.DOCX]

**Supplementary Materials**

Supplementary Table 1. SynaG Study protocol for different schedules. P = parent; C= child, A = adult, S = self-report, * = PMS cohort only, ** = NRXN1 del and TD cohort only.

|  | **Measures** | **Schedule A**(estimated VMA < 24 months) | **Schedule B** (estimated VMA ≥24  and ≤ 60 months) | **Schedule C** (estimated VMA  >60 months) | **Schedule D****   (adult self- report schedule) |
| --- | --- | --- | --- | --- | --- |
| **Clinical diagnosis and symptomatology** | ADI‑R (if MA > 18months) | P | P | P | - |
|  | ADOS‑2 (Modules 1-3 or Toddler Module depending on age and ability level) | C | C | C | - |
|  | Aberrant Behavior Checklist (ABC) | P | P | P | S |
|  | Childhood Routines Inventory-Revised (CRI-R) | P | P | P | - |
|  | Adulthood Routines Inventory-Revised (ARI-R) | P | P | P | S |
|  | Short Sensory Profile (SSP) | P | P | P | - |
|  | Sensory Experience Questionnaire (brief version 3.0) | P | P | P | - |
|  | DSM-IV and DSM-5 ASD checklists (clinician-rating) | P | P | P | S |
|  | Social Communication Questionnaire (SCQ) | P | P | P | S |
|  | Social responsiveness scale (SRS) | - | - | - | S |
|  | Autism Quotient (AQ) | - | - | - | S |
| **Adaptive behaviour** | Vineland Adaptive Behaviour Scale‑II | P | P | P | P |
|  | Columbia Impairment Scale | - | - | P | S |
|  | Developmental Coordination Disorder Questionnaire (DCDQ) | P | P | P | - |
| **Comorbidities** | Early/ Child Social Behaviour Questionnaire | P | - | - | - |
|  | Adult Social Behaviour Questionnaire | - | - | - | S |
|  | Reiss Scales for Children's Dual Diagnosis | P | P | P | - |
|  | The Psychiatric and Schizotypal Inventory for children (PSI-C / CO-LIFE) | P | P | P | S |
|  | Beck’s Anxiety/Depression Inventories | - | - | - | S |
|  | DSM‑5 ADHD Rating Scales | - | - | - | S |
| **Background measures** | Demographics | P | P | P | S |
|  | Family medical history, background | P | P | P | S |
|  | Medical history | P | P | P | S |
|  | Pre‑perinatal history | P | P | P | - |
|  | Sleep Questionnaire | P | P | P | - |
|  | Family History Interview (FHI) | P | P | P | S |
| **General Intellectual Ability and Cognitive Function** | Mullen Scales of Early Learning (MSEL) | C | C | - | - |
|  | British Picture Vocabulary Scale: Third Edition (BPVS) / Raven’s Coloured Progressive Matrices (CPM) | - | - | C | - |
|  | British Ability Scales (BAS) | - | - | C | - |
|  | Wechsler Abbreviated Scale of Intelligence - Second edition (WASI-II) |  |  | C/A | A |
| **Social Cognition*** | Parent‑Child Interaction (modules 1-3 depending on age/ability levels)* | C+P | C+P | C+P | - |
|  | Social orienting* | C | C | - | - |
|  | Penny Hiding Game* | - | C | C | - |
| **Eye‑tracking** | Natural viewing of social scenes: static and dynamic | C | C | C/A | A |
|  | Gap attention shifting | C | C | C/A | A |
|  | Biological motion | C | C | C/A | A |
|  | Implicit false belief | C | C | C/A | A |
| **EEG** | Auditory Mismatch negativity | C | C | C/A | A |
|  | Social / non-social videos task | C | C | C/A | A |
|  | Auditory gamma | C | C | C/A | A |
|  | Face ERP** | C | C | C/A | A |
|  | Resting state** | C | C | C/A | A |
| **MRI/ MRS** | Structural ADNI Accelerated | C | C | C/A | A |
|  | DTI | C | C | C/A | A |
|  | MRS (GABA, glutamate) | C | C | C/A | A |
| **Biological samples** | Blood sample(+ both parents) | C | C | C/A | A |
|  | Saliva sample (+ both parents) | C | C | C/A | A |
|  | Hair sample (+ both parents)* | C | C | C/A | A |
|  | Head circumference, height, weight. | C | C | C/A | A |
| **Parental measures** | Matrix Adaptive IQ test/ WASI II | P | P | P | - |
|  | Adult Routines Inventory | P | P | P | - |
|  | Social Responsiveness Scale (adult about self) | P | P | P | - |
|  | Beck’s Anxiety/Depression Inventories | P | P | P | - |
|  | Autism Quotient | P | P | P | - |
|  | DSM‑5 ADHD Rating Scales | P | P | P | - |
|  | Oxford-Liverpool Inventory of Feelings and Experiences (O-LIFE) | P | P | P | - |

Supplementary Table 2. Current task completion rates. The highest rates of overall task completion thus far are highlighted in green and the lowest in yellow/red.

|  | PMS (N=25) |  | NRXN1ds (N=36*) |  | iASD (N=33) |  | TD-PMS (N=30) |  | TD-NRXN (N=22) |  | **Total (N=146)** |  |
| --- | --- | --- | --- | --- | --- | --- | --- | --- | --- | --- | --- | --- |
|  | n | % | n | % | n | % | n | % |  | % | **n** | **%** |
| **Autism features**** |  |  |  |  |  |  |  |  |  |  |  |  |
| ADOS | 21 | 84% | 7 | 19% | 30 | 91% | - | - | - | - | **63** | **67%** |
| ADI-R | 25 | 100% | 10 | 28% | 28 | 85% | - | - | - | - | **68** | **72%** |
| **Comorbidities and background** |  |  |  |  |  |  |  |  |  |  |  |  |
| DAWBA | - | - | 25 | 69% | - | - | - | - | - | - | **25** | **74%** |
| Online questionnaires | 14 | 56% | 10 | 28% | 26 | 79% | 18 | 60% | 0 | 0% | **70** | **48%** |
| **Cognition and adaptive functioning** |  |  |  |  |  |  |  |  |  |  |  |  |
| Cognitive assessment/ IQ | 22 | 88% | 22 | 61% | 30 | 91% | 30 | 100% | 21 | 95% | **130** | **89%** |
| CANTAB | - | - | 21 | 58% | - | - | - | - | 21 | 95% | **42** | **75%** |
| Vineland-II (VABS) | 24 | 96% | 8 | 22% | 29 | 88% | 28 | 93% | 0 | 0% | **94** | **64%** |
| **Social Cognition** |  |  |  |  |  |  |  |  |  |  |  |  |
| Social Orienting task (SO) | 18 | 72% | - | - | 25 | 76% | 29 | 97% | - | - | **76** | **86%** |
| Penny-Hiding Game (PHG) | 7 | 28% | - | - | 20 | 61% | 23 | 77% | - | - | **52** | **59%** |
| Parent-Child Interaction (PCI) | 20 | 80% | - | - | 30 | 91% | 30 | 100% | - | - | **85** | **97%** |
| Eye-tracking (ET) | 18 | 72% | 3 | 8% | 23 | 70% | 30 | 100% | 2 | 100% | **80** | **55%** |
| **Neurocognition, brain structure and function** |  |  |  |  |  |  |  |  |  |  |  |  |
| EEG | 21 | 84% | 2 | 6% | 16 | 48% | 22 | 73% | 1 | 50% | **64** | **44%** |
| MRI | 4 | 16% | 17 | 47% | 6 | 18% | 6 | 20% | 17 | 77% | **50** | **34%** |
| **Biosampling** |  |  |  |  |  |  |  |  |  |  |  |  |
| Bloods | 19 | 76% | 3 | 8% | 7 | 21% | 1 | 3% | 0 | 0% | **31** | **33%** |
| Saliva | 15 | 60% | 2 | 6% | 26 | 79% | 23 | 77% | 0 | 0% | **68** | **47%** |
| Hair | 21 | 84% | 2 | 6% | 3 | 9% | 1 | 3% | - | - | **28** | **30%** |
| Skin biopsy | - | - | 11 | 31% | - | - | - | - | - | - | **11** | **32%** |

| *2 NRXN1 dels participants not assessed using DAWBA, CANTAB, or skin biopsy measures. |
| --- |
| **Not assessed in TD comparison groups. |

| **Box 1. Measures taken to facilitate study engagement.**  **Anxiety management strategies**   - To help reduce anxiety around uncertainty of the research visit, picture books including photos of rooms, spaces, and people the participant will encounter are sent to families prior to a visit. - Sounds from MRI scan sequences are shared with families before the research visit so that participants can practice laying still whilst listening to the sounds at home.   **Behavioural strategies**   - Families are encouraged to share any potential triggers or warning signs, and useful strategies with the research team to facilitate research visits. - Modelling behaviours and positive behavioural support strategies are used consistently to facilitate understanding and to enhance likelihood of engagement. - During research visits, parent/caregiver expertise in their children are utilised to facilitate setting up participants for EEG and the MRI scan. - Families are encouraged to bring the participant’s favourite object/toy (a transitional object) to the research visit to provide comfort and motivation, and their favourite DVD to be played during sections of the EEG recording if they wish. - Breaks are encouraged throughout research visits to facilitate participants with limited attention span or difficulties sitting still for long periods of time. - A sleep scan option using melatonin has also been introduced at KCL for participants who may not complete the scan session whilst awake.   **Desensitisation strategies**   - Researchers at KCL have created a space journey narrative around the MRI scanning process, whereby undergoing the MRI scan is likened to a space journey inside a rocket. - The sounds of the scanner can therefore be thought of as rocket sounds and the journey involves exploring one’s brain. A specially designed ‘space tent’ of the night sky stands around the scanner to somewhat veil the clinical surroundings and the participant has the opportunity to meet ‘space characters’, which are soft toys they can place on the space tent using Velcro (see supplementary Figure 2). - Prior to entering the MRI environment, each participant has the opportunity to practice the procedure using a ‘mock’ deactivated scanner environment and a smaller yet identical space tent. Incremental practice of the MRI procedure within a ‘mock scanner’ environment is available to participants who may find it beneficial. |
| --- |

Supplementary Figure 1. Rationale for study schedule assignment of cognitive ability assessment.


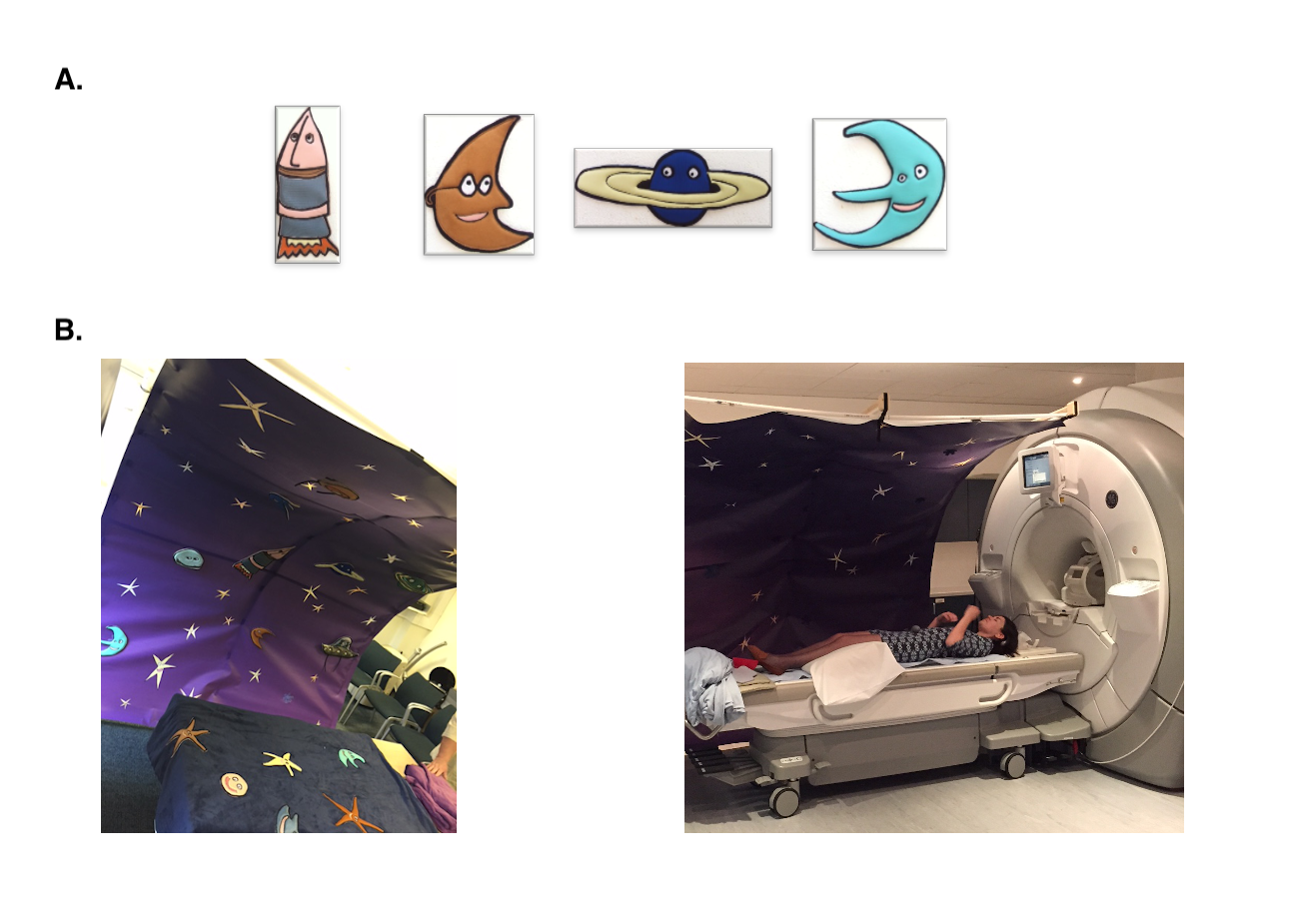


Supplementary Figure 2. MRI Space Story equipment and set-up to facilitate MRI scans in children at KCL. A. Space ‘characters’ that participants are introduced to before and during the MRI visit; B. Set up of MRI machine with space tent and space characters.
